# Forest Management Intensity Affects Aquatic Communities in Artificial Tree Holes

**DOI:** 10.1371/journal.pone.0155549

**Published:** 2016-05-17

**Authors:** Jana S. Petermann, Anja Rohland, Nora Sichardt, Peggy Lade, Brenda Guidetti, Wolfgang W. Weisser, Martin M. Gossner

**Affiliations:** 1 Department of Ecology and Evolution, University of Salzburg, Salzburg, Austria; 2 Berlin-Brandenburg Institute of Advanced Biodiversity Research (BBIB), Berlin, Germany; 3 Institute of Ecology, Friedrich-Schiller-University, Jena, Germany; 4 Terrestrial Ecology Research Group, Department of Ecology and Ecosystem Management, Center for Food and Life Sciences Weihenstephan, Technische Universität München, Freising-Weihenstephan, Germany; DOE Pacific Northwest National Laboratory, UNITED STATES

## Abstract

Forest management could potentially affect organisms in all forest habitats. However, aquatic communities in water-filled tree-holes may be especially sensitive because of small population sizes, the risk of drought and potential dispersal limitation. We set up artificial tree holes in forest stands subject to different management intensities in two regions in Germany and assessed the influence of local environmental properties (tree-hole opening type, tree diameter, water volume and water temperature) as well as regional drivers (forest management intensity, tree-hole density) on tree-hole insect communities (not considering other organisms such as nematodes or rotifers), detritus content, oxygen and nutrient concentrations. In addition, we compared data from artificial tree holes with data from natural tree holes in the same area to evaluate the methodological approach of using tree-hole analogues. We found that forest management had strong effects on communities in artificial tree holes in both regions and across the season. Abundance and species richness declined, community composition shifted and detritus content declined with increasing forest management intensity. Environmental variables, such as tree-hole density and tree diameter partly explained these changes. However, dispersal limitation, indicated by effects of tree-hole density, generally showed rather weak impacts on communities. Artificial tree holes had higher water temperatures (on average 2°C higher) and oxygen concentrations (on average 25% higher) than natural tree holes. The abundance of organisms was higher but species richness was lower in artificial tree holes. Community composition differed between artificial and natural tree holes. Negative management effects were detectable in both tree-hole systems, despite their abiotic and biotic differences. Our results indicate that forest management has substantial and pervasive effects on tree-hole communities and may alter their structure and functioning. We furthermore conclude that artificial tree-hole analogues represent a useful experimental alternative to test effects of changes in forest management on natural communities.

## Introduction

Human management affects forests in many ways. Direct impacts include changes in forest structure such as increased densities of trees with small crown sizes, more homogeneous tree ages and lower tree diversity in intensively managed forests compared with less intensively managed ones [1, 2]. Indirect effects of forest management include altered light levels, higher disturbance and changes in plant and animal communities [1, 3–5]. In central Europe almost all forests are managed to some extent [6]. However, the effects of different management intensities especially on forest-dwelling small organisms such as arthropods are still underexplored.

Forests provide a large number of different habitats. One habitat that has received little attention are water-filled tree holes. These holes form for various reasons, for example as a result of branch breaks or woodpecker activity and often fill up with water [7, 8]. Different types of holes such as pans (with a bark lining) and rot holes (no bark lining, direct contact with rotting wood) can be distinguished ([7, 8], for pictures of natural tree holes in our study region see S1 Fig). Arthropods that require aquatic habitats for part of their life cycle colonize these tree holes and typically spend their larval stages there [7, 8]. These are predominantly insect species of Diptera such as Ceratopogonidae, Chironomidae, Culicidae, Cecidomyiidae, Muscidae, Psychodidae and Syrphidae and Coleoptera such as Scirtidae [9]. In addition, crustaceans, nematodes, rotifers, protozoa and bacteria may be part of tree-hole communities (however, we do not consider these groups here). Few of the species in tree-hole communities are considered habitat specialists that depend exclusively on tree holes (dendrolimnetobionts [7, 8]). Nevertheless, water-filled tree holes may, even for habitat generalists, constitute important aquatic breeding sites which—depending on the general or temporary availability of other water sources—may be rare in forests. In a previous study, we found that the density of natural water-filled tree holes may vary from zero to up to 50 or more per ha in German forests [10]. The arthropod species colonizing tree holes are mostly detritus feeders and consume dead leaves and other dead plant material accumulating in the tree holes. Predators are surprisingly rare in tree-hole communities in Europe [9]. The communities that are formed in tree holes are classical examples of metacommunities [11, 12]. Here, local tree-hole inhabiting larval communities are linked by the dispersal of adult life stages [8]. Tree-hole communities are a suitable system to investigate metacommunity dynamics and, more generally, processes that drive community structure [13, 14], especially because they are small and limited in the species richness and population sizes of the inhabiting organisms [15]. However, small population sizes and the dependence on detrital resources, which are often limited [16–18], as well as potential dispersal limitation [19, 20] may make tree-hole communities prone to strong impacts of environmental change. Therefore, in addition to their use as a model system for testing fundamental ecological questions, they may represent important indicators of subtle forest management effects as they may be more sensitive to changes than larger arthropod communities.

A number of abiotic factors and forest structural attributes have been shown to affect aquatic tree-hole communities. The communities, or at least some of their species, may be sensitive to drought, which occurs more frequently in shallow compared with deep holes [21]. Drought and temperature extremes might also partly explain the strong seasonal fluctuations of species colonizing artificial tree holes found by Ptatscheck and Traunspurger [22]. Tree-hole communities may therefore be influenced by the volume and shape of the tree hole which will affect the probability of drying out and its detritus content, the basic resource of these bottom-up regulated systems. However, many effects have been found to be species- or functional group-specific [9, 23]. Tree-hole inhabiting species may be influenced by other habitat features in the surrounding forest that may limit their occurrence in the regional species pool. For example, some species need terrestrial food sources or shelter during their adult life stages [24]. Typically, adults have to disperse through the terrestrial matrix between tree holes [25], which may be an important factor limiting the colonization of tree-hole habitats.

Even though tree-hole communities have been shown to be affected by forest attributes that may be modified by management, few studies have directly investigated the effect of management on the structure and functioning of tree-hole communities. A study in the tropics found that tree-hole community composition in forests under different management indeed differed [26]. For example, top predator species identity shifted from damselfly larvae to a predatory mosquito species when forest was converted to small plantations. Artificial tree holes set up in landscape types with stronger human influence in New Zealand were colonized by macroinvertebrate communities at higher densities, but with lower species richness and different composition compared with less-influenced landscapes [27]. In another study in Germany we found that more intensively managed forests contained a lower density of natural tree holes and a lower diversity of tree-hole types which, in combination with differences in resource input and chemical water parameters, affected the structure of their inhabiting communities [10].

Nevertheless, the multitude of environmental factors that change with forest management intensity make it difficult to assign causal mechanisms to the detected effects in observational studies. Therefore, artificial tree-hole analogues may be useful experimental systems allowing the standardization of certain environmental conditions [28]. However, there are likely differences in abiotic conditions between artificial and natural tree holes which might lead to different communities colonizing these tree holes [28] and thus to different effects of the environment and of experimental treatments. In this study, we use such an experimental approach with artificial tree holes, standardizing local habitat (tree-hole) size, initial water volume and initial detritus content in order to test the effects of forest management on the structure and functioning of tree-hole communities. Specifically, we measure the influence of local environmental properties (tree diameter, tree-hole opening type, water volume and water temperature) as well as regional drivers that might integrate over local ones or affect dispersal through the matrix (forest management intensity, tree-hole density). We also investigate a number of parameters related to decomposition processes in the tree holes to assess the effect of management on decomposition as the most important ecosystem function in tree holes. With two separate experiments in two regions and two sampling periods (in one region) we furthermore assess the consistency of effects under different conditions, with potentially different species pools and across the season. In addition, we compare data from artificial tree holes with data from natural tree holes in the same area to evaluate the methodological approach of using tree-hole analogues.

We hypothesize that (1) forest management intensity affects the abundance, richness and composition of local communities as well as detritus content, oxygen and nutrient concentration in water-filled tree holes, (2) local environmental conditions and dispersal limitation (indicated by effects of natural tree-hole density) have a direct influence on tree-hole communities and may explain part of the management effect, and (3) environmental conditions and arthropod communities in artificial tree holes differ from those in natural ones, however, (4) forest management effects are consistently detectable in both, artificial and natural tree holes.

## Methods

**Fig 1:**
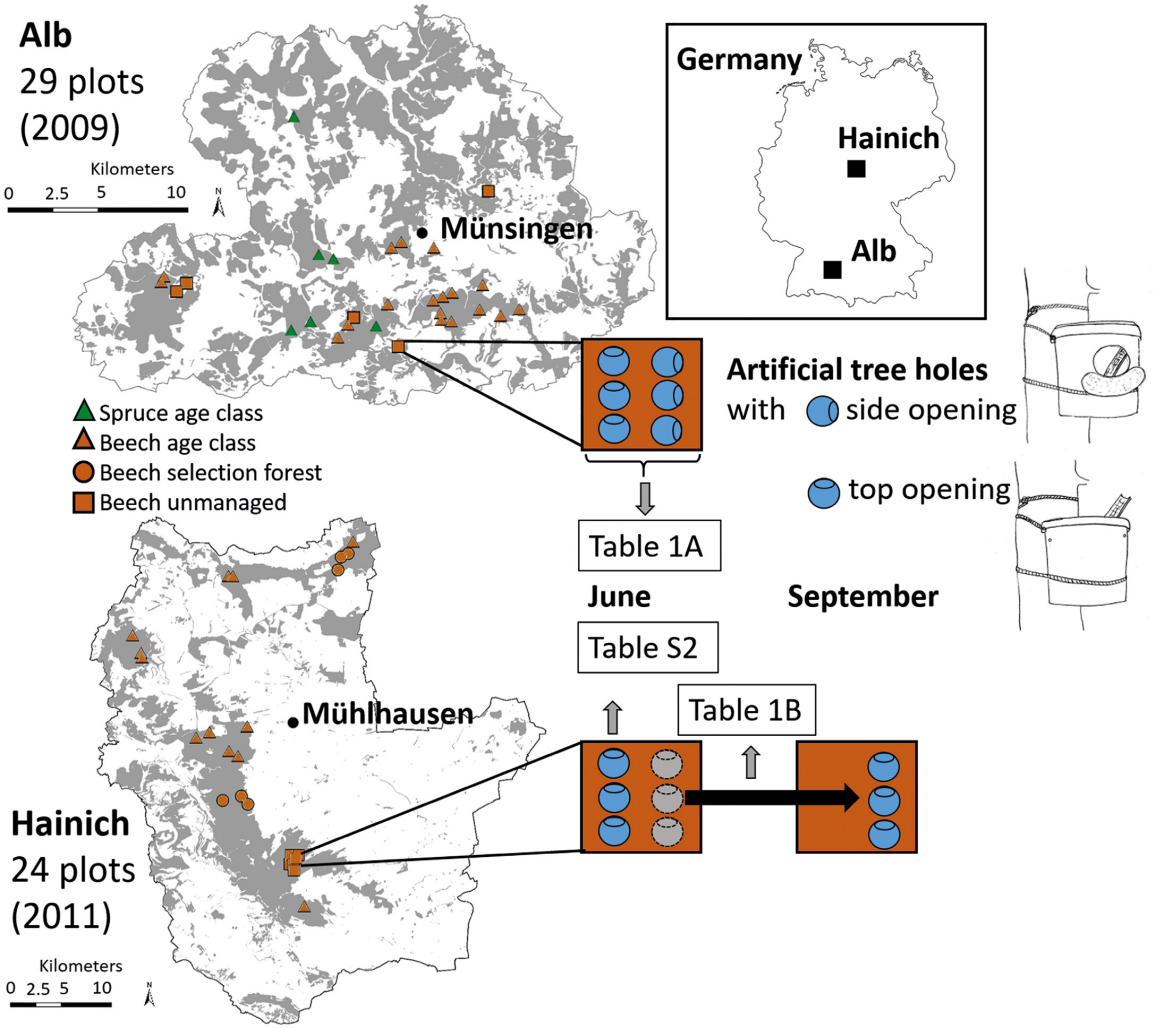
Experimental design. In the two regions (Alb and Hainich), we set up six artificial tree holes in each of 29 and 24 plots, respectively. In the Alb, three of the holes in each plot had openings at the side, three at the top. In the Hainich, all tree holes had top openings. Tree holes were installed in April. In the Alb, all tree holes were collected in June, in the Hainich, three were harvested in June, three remained until September. Tree holes in the Alb had a maximum volume of 10l, in the Hainich a substantially lower maximum volume of 600ml. For further information on tree-hole characteristics and measurements in the two regions, see S1 Table and S2 Fig. Table numbers in the figure refer to tables with the respective statistical analyses for the data sets: Table 1A and 1B for data sets involving June data in the Alb and June and September data in the Hainich, respectively, S2 Table for June data (and additional environmental variables) in the Hainich. Grey areas depict forests, white areas non-forest habitats. Maps reprinted with permission from Landesamt für Geoinformation und Landentwicklung Baden-Württemberg (17.03.2016, Az.: 2851.2-D/7537 www.lgl-bw.de) for the Alb and from GeoBasisDE / TLVermGeo (2016) for the Hainich.

This project was conducted in the framework of the Biodiversity Exploratories in Germany (www.biodiversity-exploratories.de). Experiments were set up in two of the three Biodiversity Exploratory regions (Fig 1): the Biosphere reserve “Schwäbische Alb” (henceforth “Alb”) and the National Park Hainich with the surrounding Hainich-Dün forest (henceforth “Hainich”). The Alb region is located in southwestern Germany (48°34’-48°53’N; 9°18’-9°60’E) at an elevation between 460 and 860m above sea level. It has a mean annual temperature of 6.0–7.0°C and a mean annual precipitation of 700–1000mm. The Hainich region is located in central Germany (50°94’-51°38’N; 10°17’-10°78’E), at 285 to 550m above sea level and has a mean annual temperature of 6.5–8.0°C and a mean annual precipitation of 500–800mm. Field work permits were issued by the responsible state environmental offices of Baden-Württemberg (for the Alb) and Thüringen (for the Hainich).

The Biodiversity Exploratories consist of 50 1-ha experimental forest plots of 100m x 100m in each region [29]. The forests of the Hainich site are dominated by broadleaf trees; conifers comprise only 12% of the total area. The Alb forests are dominated by *Fagus sylvatica* (46%) and *Picea abies* (24%). For more information on tree species composition in the two regions, see Boch et al. [3]. The forest plots represent a number of different management types. In selection cutting beech forests trees are harvested individually resulting in a mixed age structure. These forests occur only in the Hainich. Age-class forests are harvested using small clear cuts in spruce stands (Alb only) and thinning and clearing after natural regeneration has established in beech stands (shelterwood system; Alb and Hainich). “Unmanaged” beech forests are defined as those forests not currently under forest management. They were used either as forest pastures (Alb) or as military training areas (Hainich) in the past and were taken out of forest management 20–70 years ago.

We quantified forest management intensity using the Forest Management Intensity Index by Kahl and Bauhus (ForMI [30]) which is based on the proportion of harvested tree volume, the proportion of non-native tree species and the proportion of dead wood showing signs of saw cuts (for pictures of selected plots along the forest management intensity gradient see S3 and S4 Figs). ForMI can thus assume values between 0 (minimum forest management intensity) and 3 (maximum forest management intensity). Typical coniferous forests in the two regions showed ForMI values around 2 while beech forests showed values around 1.2 [30]. ForMI values in our plots ranged from 0 to 2.83 in the Alb and from 0 to 1.89 in the Hainich. We additionally ran all models with a further index, the Silvicultural Management Intensity Indicator (SMI [31]), to assess the robustness of our results. This index combines information on tree species, stand age and aboveground, living and dead wood biomass into a component describing the risk of stand loss dues to natural hazards (wind throw, beetle attack), while an additional component describes relative stand density. Both components of this index reflect management decisions.

### Experiment in the Alb region

We set up artificial tree holes in plots of different management intensities to test the effects of forest management on the structure of tree-hole communities colonizing these artificial tree holes. In the Alb region, we set up our experiment in 29 plots in 2009 (Fig 1, for pictures of selected plots along the forest management intensity gradient see S3 Fig). To assess the effects of potential colonizer sources we mapped natural tree holes in the plots using binoculars and a telescopic pole with a camera attached to the top. This device allowed us to map all tree holes up to a height of 17m. Only tree holes with a maximum volume of larger than 50ml were used to calculate the density of tree holes per 1-ha plot. Other potential aquatic habitats in the plots were not recorded. However, these were only very few small puddles and water-filled tracks of forestry machinery. As artificial tree holes we used white cylindrical containers with a lid, an upper diameter of 272mm, a lower diameter of 230mm, a height of 256mm and a total volume of 10l (Figs 1 and S2). In comparison, natural tree holes in the region had an estimated maximum volume of 1000.5±192.2ml, ranging from 5ml (lower sampling limit) to more than 16.5l. Six containers were installed at the border of the 20m x 20m core area in each of the 29 plots on the southwestern side of trees (n = 174) at a distance of about 10m. Most containers were attached to European beech (*Fagus sylvatica*) except for containers in spruce stands which were set up at spruce trees (*Picea abies*). We tested two different opening types of the artificial tree holes. Three containers were open at the top to mimic tree-hole types such as pans and rot holes, while the other three containers had an opening of a diameter of 95mm drilled into the side mimicking woodpecker holes (Fig 1). Containers had drainage holes just below the maximum water level to prevent them from overflowing after heavy rains. Containers were directly attached to trees, so top-opening types could potentially receive input of organic material and organisms through stemflow. Water levels were checked regularly to make sure that containers did not fall dry.

Artificial tree holes received an initial standardized input of dead leaves of 20g dry weight as a detritus resource for colonizing species. These leaves of mixed species origin were collected in a deciduous forest outside the plots and dried at 70°C twice for eight hours. This detritus amount resulted in an uncompressed detritus volume of about 2500ml or 50% of the maximum water volume following Yanoviak and Fincke [28]. Detritus was manually ground to include coarse and fine detritus fractions. Artificial tree holes were filled with 2.5l of rainwater from the region. We also added a wooden board (30cm x 3cm) and attached a piece of fabric to the wall of each tree hole to facilitate insect oviposition [32]. In mid-April, the tree holes were attached with plastic ropes to the southwestern side of trees at a height of about 1.8m (Fig 1). In June, after an experimental duration of six weeks, we measured the water volume and collected the contents of the artificial tree holes. In each plot, two subsamples, one from an artificial hole with an top opening and one from an artificial hole with a side opening, were frozen to determine the ammonium content photometrically in the laboratory to evaluate decomposition processes in the tree holes (for details see S1 Methods and for an overview of tree-hole characteristics and measurements see S1 Table).

### Experiment in the Hainich region

In the Hainich region, we mapped natural tree holes in the same way as described above and installed our artificial tree holes in 24 plots in April 2011 (Fig 1, for pictures of selected plots along the forest management intensity gradient see S4 Fig). Six tree holes were set up in each plot (n = 144). As artificial holes we used cylindrical plastic containers with an upper diameter of 115mm, a lower diameter of 85mm, a height of 163mm and a total volume of 1l, with two drainage holes at the 600-ml mark (S2 Fig). Natural tree holes in the region had an estimated maximum volume of 536.2±72.4ml, ranging from 5ml (lower sampling limit) to more than 10l. The artificial holes received 500ml of rainwater from the region and 1.2g beech leaves as detritus that was collected from a nearby beech-dominated forest, dried at 70°C twice for eight hours and ground by hand. This detritus amount corresponds to 150ml or 25% of the maximum water volume [28]. Furthermore, we added wooden boards (beech, 18mm x 150mm x 9mm) as oviposition structures. The artificial tree holes were attached with a rope at a height of 1.8m to the southwestern side of trees with different diameters at the border of a 20m x 20m core area in a distance of about 10m. Almost all artificial tree holes were attached to European beech (*Fagus sylvatica*) except three containers which were attached to ash (*Fraxinus excelsior*) in one plot where too few beech trees occurred around the core area. Containers were directly attached to the bark of the trees, so stemflow could augment the input of organic material and organisms into the artificial tree holes. Water levels were checked regularly to make sure that containers did not fall dry. During a dry period at the end of April we had to refill almost all artificial tree holes in this region with 300ml of rainwater (except for five holes that had more than 500ml of water remaining). Five holes had already fallen dry at this time and were refilled.

After an experimental duration of nine weeks (in June), we sampled half of the tree holes in each plot, the rest of the tree holes were sampled after five months (in September) to assess effects of season on tree-hole communities and detritus content. Oxygen content was measured with a mobile electrode (WTW GmbH, Oxi 330) as a process associated with decomposition processes. We also measured water temperature and pH (WTW GmbH, pH 330) and then took a 15-ml water sample using a syringe with a 0.45-μm filter (PVDF diaphragm). This sample was cooled in the field and later frozen at -20°C. The contents of the tree hole were removed and taken to the laboratory for the identification of the contained organisms. In the laboratory, we measured nitrate (NO_3_^-^), ammonium (NH_4_^+^), and phosphate (PO_4_^3-^) concentrations photometrically on the thawed water samples as further variables related to decomposition (for details see S1 Methods and for an overview of tree-hole characteristics and measurements see S1 Table). Detritus from each artificial tree hole was dried at room temperature and detritus volume was measured according to Yanoviak and Fincke [28].

### Species identification

All aquatic developmental stages of insects (larvae, pupae) were preserved in 70% ethanol and identified under the microscope to the highest taxonomic resolution possible using Cranston [33], Lindegaard [34] and Pankratowa [35] for Chironomidae, Nilsson [36] for Ceratopogonidae, Cranston et al. [37], Mohrig [38] and Utrio [39] for Culicidae, Rozkošný and Gregor [40] for Muscidae, Dixon [41], Hartley [42], and Rotheray [43] for Syrphidae, Nilsson [36] for Psychodidae, Nilsson [36] for Tipulidae and Klausnitzer [44] for Scirtidae. Many of the insect larvae could not be identified to species level and they were classified into morphospecies.

### Data analysis

All analyses were carried out in R version 3.1.2 [45]. With linear mixed effects models using the function lme in R package nlme [46] we analyzed the effect of (1) forest management intensity, (2) the density of natural tree holes (as a proxy for dispersal opportunities) as well as (3) the environmental parameters (a) diameter of the tree that hosted the artificial tree hole as an additional descriptor of the potential quality of the local habitat, e.g. as shelter for adults (Hainich only), (b) tree-hole opening type (Alb only), (c) water volume at the end of the experiment (Alb only) and (d) water temperature at the end of the experiment (Hainich, June only) on the abundance and species richness of tree-hole communities as well as detritus content (volume, Hainich only), phosphate (Hainich, June only), nitrate (Hainich, June only) and ammonium content, and oxygen concentration (Hainich June only) in the water. Abundance data were square-root transformed and phosphate, nitrate, ammonium content and oxygen concentration were log-transformed to meet the assumptions of the models. All two-way interactions with forest management intensity and month in which samples were collected (June or September) were included in the models. We additionally fit mixed effects models with different spatial autocorrelation functions (exponential, Gaussian, linear spatial, rational quadratics and spherical). There was no difference between the models with and without spatial correlation structure, so we present results from models without autocorrelation structure. To test if the effects of forest management intensity on abundance and richness operated via changes in environmental variables, we ran additional models in which we tested the effect of forest management intensity after all main effects in the model.

To visualize differences in the insect community compositions of artificial tree-hole communities we used non-metric multidimensional scaling (NMDS) plots produced with the metaMDS function in R package vegan [47] with a maximum of 100 random starts and two dimensions. PERMANOVAs on Bray-Curtis matrices with 9999 permutations (function adonis in vegan) were run to test the effect of forest management intensity, density of natural tree holes as well as hole opening type and water volume (Alb only) on community composition.

To assess the use of artificial containers in tree-hole studies, we compared our data to data from a study in natural tree holes conducted in the same forests and in the same years [10]. We compared environmental parameters (water temperature and oxygen concentration), abundance and richness of the communities. To illustrate differences in community composition between artificial and natural tree holes we used an NMDS plot and PERMANOVAs (see above).

## Results

In total, we found 27,269 individuals of 37 (morpho-) species of Diptera and of one species of Coleoptera in the artificial tree holes (for a species list see S5 Fig). Twenty-four artificial tree holes (20 tree holes or 11% in the Alb, 4 tree holes or 3% in the Hainich) did not contain any insect larvae.

### Abundance and species richness

**Fig 2:**
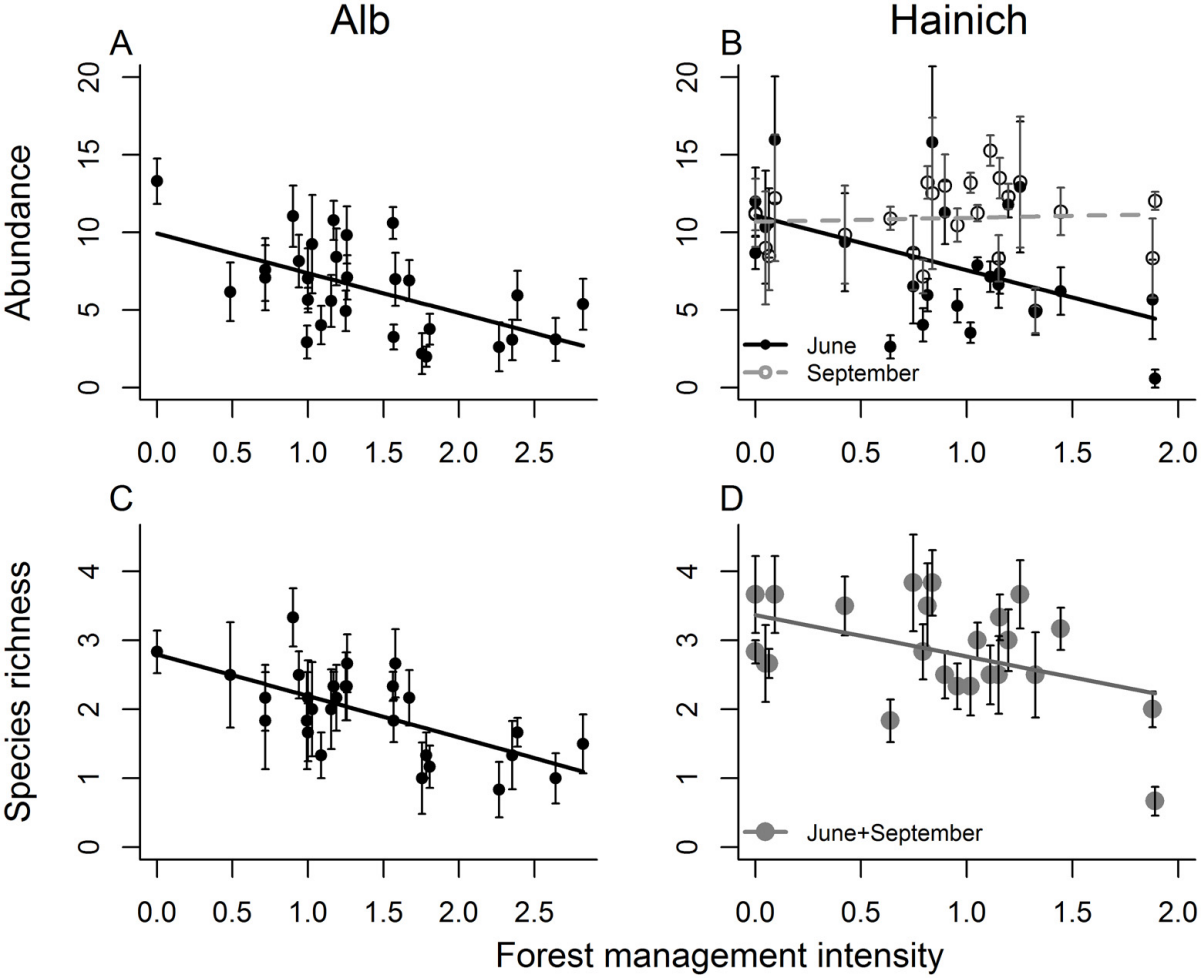
Effect of forest management intensity on abundance and species richness. Forest management intensity (according to Kahl and Bauhus [30]) effects on abundance (square-root transformed, A, B) and species richness (C,D) of macroinvertebrate communities in artificial tree holes in the Alb (A,C) and Hainich (B,D) region. Data are from June (closed symbols and solid lines) or from September (open symbols and dashed line in B) or pooled across sampling times because there was no significant interaction of forest management intensity and time (large grey symbols and grey line in D, Table 1). In the Alb, the two different opening types (top vs. side) were pooled because effects of forest management on abundance and richness did not differ between the two types (Table 1). Please note that experimental set-up and the volume of artificial tree holes varied between the two regions, so they cannot be compared directly. Symbols represent means per plot, error bars show ±SE. For results of statistical analyses see Table 1.

The abundance of organisms in artificial tree holes was negatively affected by forest management intensity in both regions in June (Fig 2A and 2B, Table 1 and S2 Table). In September (only data from the Hainich region were available) no effects of forest management were detected. In the Alb, where we tested tree holes with different opening types, holes with a top opening contained higher numbers of organisms than holes with a side opening (Table 1A). In the Hainich, the overall abundance of organisms in the holes was higher in September than in June (Fig 2B, Table 1B). Tree diameter also had an effect on abundance: the smaller the diameter of the tree that the artificial container was attached to, the higher the abundance (Table 1B). When we changed the order of the variables in the model and tested the effect of forest management intensity after all other main effects, we found that in the Alb the significant negative effect of forest management on abundance remained significant (F_1,25_ = 5.01, P = 0.0343) but that it was weaker and partly explained by a significant positive effect of tree-hole density (F_1,25_ = 8.59, P = 0.0071). In the Hainich, the effect of forest management on abundance disappeared when tested as the last main effect in the model for the full data set (F_1,20_ = 0.56, P = 0.4638) and for the June data (F_1,19_ = 2.35, P = 0.1421). In the June data it was partly explained by significant positive effects of tree-hole density and tree diameter (F_1,19_ = 8.65, P = 0.0084 and F_1,41_ = 6.33, P = 0.0159, respectively). Tree-hole density declined with increasing forest management intensity in both regions (F_1,51_ = 36.88, P<0.001 from a linear model). The average diameter of the trees that artificial tree holes were attached to declined with forest management intensity in the Hainich (F_1,22_ = 6.76, P = 0.0163 from a linear mixed model of log-transformed tree diameter with plot as a random effect).

**Table 1:** Mixed model results for abundance and richness.

		Abundance				Richness			
	ndf	ddf	F	P		ddf	F	P	
**A) Alb (June)**									
(Intercept)	1	141	185.57	<0.001		140	820.68	<0.001	
Abundance						140	98.77	**<0.001**	**↑**
Forest management intensity (ForMI)	1	25	12.42	**0.0016**	**↓**	25	5.10	**0.033**	**↓**
Tree-hole density	1	25	0.76	0.3910		25	0.51	0.4812	
Opening type	1	141	44.49	**<0.001**		140	38.89	**<0.001**	
Volume	1	141	3.21	0.0754		140	0.07	0.7916	
ForMI x Tree-hole density	1	25	2.95	0.098		25	0.40	0.5328	
ForMI x Opening type	1	141	3.09	0.0807		140	0.50	0.4792	
ForMI x Volume	1	141	1.07	0.3035		140	2.29	0.1323	
**B) Hainich (June and September)**									
(Intercept)	1	91	480.18	<0.001		90	640.28	<0.001	
Abundance						90	10.83	**0.0014**	**↑**
Forest management intensity (ForMI)	1	20	4.64	**0.0436**	**↓**	20	6.11	**0.0225**	**↓**
Tree-hole density	1	20	0.33	0.5725		20	0.48	0.4985	
Time	1	20	12.24	**0.0023**	**↑**	20	2.84	0.1073	
Tree diameter	1	91	6.36	**0.0134**	**↓**	90	3.50	0.0646	
ForMI x Tree-hole density	1	20	0.83	0.3741		20	0.27	0.6061	
ForMI x Time	1	20	5.86	**0.0252**		20	0.24	0.6327	
ForMI x Tree diameter	1	91	2.67	0.1055		90	1.30	0.2569	
Time x Tree-hole density	1	20	0.16	0.6978		20	0.39	0.5381	
Time x Tree diameter	1	91	0.02	0.8931		90	0.73	0.3957	

Results from linear mixed models testing the effect of forest management intensity and a number of environmental variables on the abundance (square-root transformed) and species richness of communities in artificial tree holes in the two regions Alb (A) and Hainich (B). Abundance (square-root transformed) is used as a covariate in the analyses of richness. Forest management intensity (ForMI) was calculated according to Kahl and Bauhus [30]. Tree-hole density describes the number of natural tree holes per plot. In the Alb, artificial tree holes with two different opening types (top vs. side) were used. Volume is the final water volume of artificial tree holes in ml. Time refers to data collection in June vs. September (September data was only available for the Hainich). Tree diameter was measured at breast height in cm. P-values<0.05 are printed in bold. For significant continuous main effects the direction of the effect is given: ↑ positive, ↓ negative. ndf: numerator degrees of freedom, ddf: denominator degrees of freedom.

Species richness was negatively affected by forest management in both regions and across time (Fig 2C and 2D, Table 1 and S2 Table). Richness was strongly related to abundance (Table 1 and S2 Table). However, the effects of forest management and other explanatory variables on richness were independent of changes in abundance (i.e. the effects were qualitatively similar with and without abundance as a covariate; results of analysis without covariate not shown). The opening type of the tree holes had an effect on richness: richness was higher in artificial tree holes with a top opening compared to a side opening. When we tested forest management intensity as the last main effect in the model, its influence on species richness disappeared in the Alb (F_1,25_ = 3.77, P = 0.0636) and in the Hainich (F_1,20_ = 0.5580, P = 0.4638). In the Hainich (but not in the Alb), it was partly explained by a positive effect of tree diameter (F_1,91_ = 7.32, P = 0.0081), which was strongly negatively affected by forest management intensity (see above). Additional models with the Silvicultural Management Intensity Indicator SMI instead of the Forest Management Intensity Index ForMI gave qualitatively very similar results for effects on abundance and richness (results not shown).

### Community composition and individual species responses

**Fig 3:**
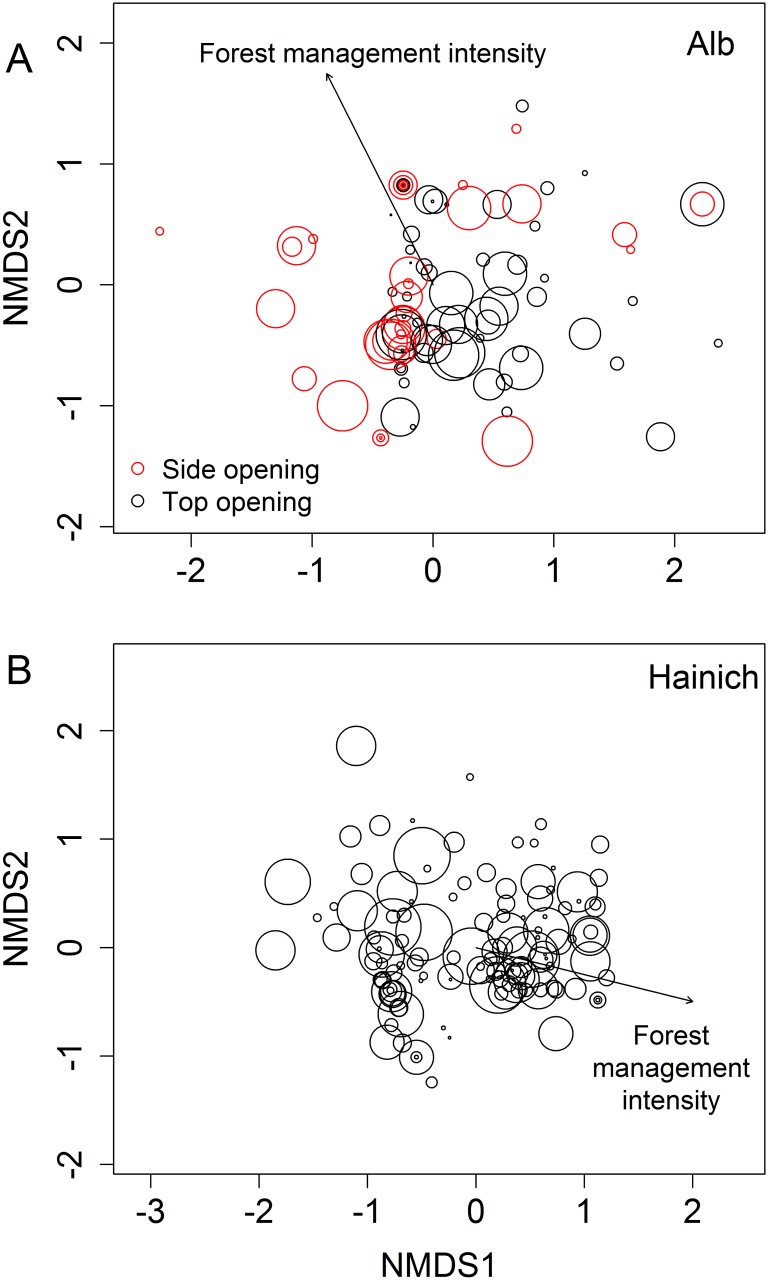
Insect community composition. Nonmetric multidimensional scaling (NMDS) plot showing the composition of insect communities in artificial tree holes in the two regions in June: A) Alb, B) Hainich. Red symbols in A represent artificial holes with a side opening, symbols in black are artificial tree holes with a top opening. The size of the symbols represents the density of natural tree holes in the respective plots. Forest management intensity affected community composition in the Alb and in the Hainich as shown by the arrow drawn by fitting forest management intensity *post hoc* and indicating tree holes in plots with high and low forest management. Opening type and tree-hole density had an effect on community composition in the Alb only (shown by the clustering of similar symbols). For results of statistical analyses, see text. Only taxa with more than five occurrences per region across both tree-hole types were used for the plots. Stress = 0.16, n = 137 for Alb, stress = 0.19, n = 134, for Hainich.

Forest management intensity had an effect on community composition in the Alb (Fig 3, F_1,132_ = 7.06, P<0.001) and in the Hainich (F_1,131_ = 2.70, P = 0.0220). Furthermore, community composition was affected by tree-hole density in the Alb (F_1,132_ = 2.74, P = 0.0240) but not in the Hainich (F_1,132_ = 0.37, P = 0.8940).

Opening type (F_1,132_ = 5.72, P<0.001) had an effect on community composition in the Alb (not studied in the Hainich), while water volume at the time of sampling did not have an effect in the Alb (F_1,132_ = 1.00, P = 0.3960, not measured in the Hainich).

Four taxa were on average much more abundant than the other species in the artificial tree holes: *Dasyhelea* sp., *Metriocnemus cavicola*, an unidentified Cecidomyiidae species (Cecidomyiidae sp. 1) and *Myathropa florea* (S5 Fig). The individual abundances of these four taxa were not influenced by forest management (S5 Table). However, the abundance of one of the four taxa (Cecidomyiidae sp. 1) responded positively to tree-hole density in the Alb and showed a significant interaction between forest management intensity and temperature in the Hainich in June (F_1,28_ = 7.48, P = 0.0107). Another one of the four most abundant species, *Dasyhelea* sp, also exhibited a marginal positive effect of tree-hole density, but only for the June data (F_1,12_ = 5.00, P = 0.0451). *M*. *florea* was more abundant in artificial tree holes with a top opening than with a side opening. The abundances of all of the four abundant taxa differed strongly between the months in the Hainich: *Dasyhelea* sp. and *M*. *cavicola* were more abundant in September, Cecidomyiidae sp. 1 and *M*. *florea* were more abundant in June (S5 Fig, S5 Table).

### Detritus content, oxygen and nutrient concentrations

**Fig 4:**
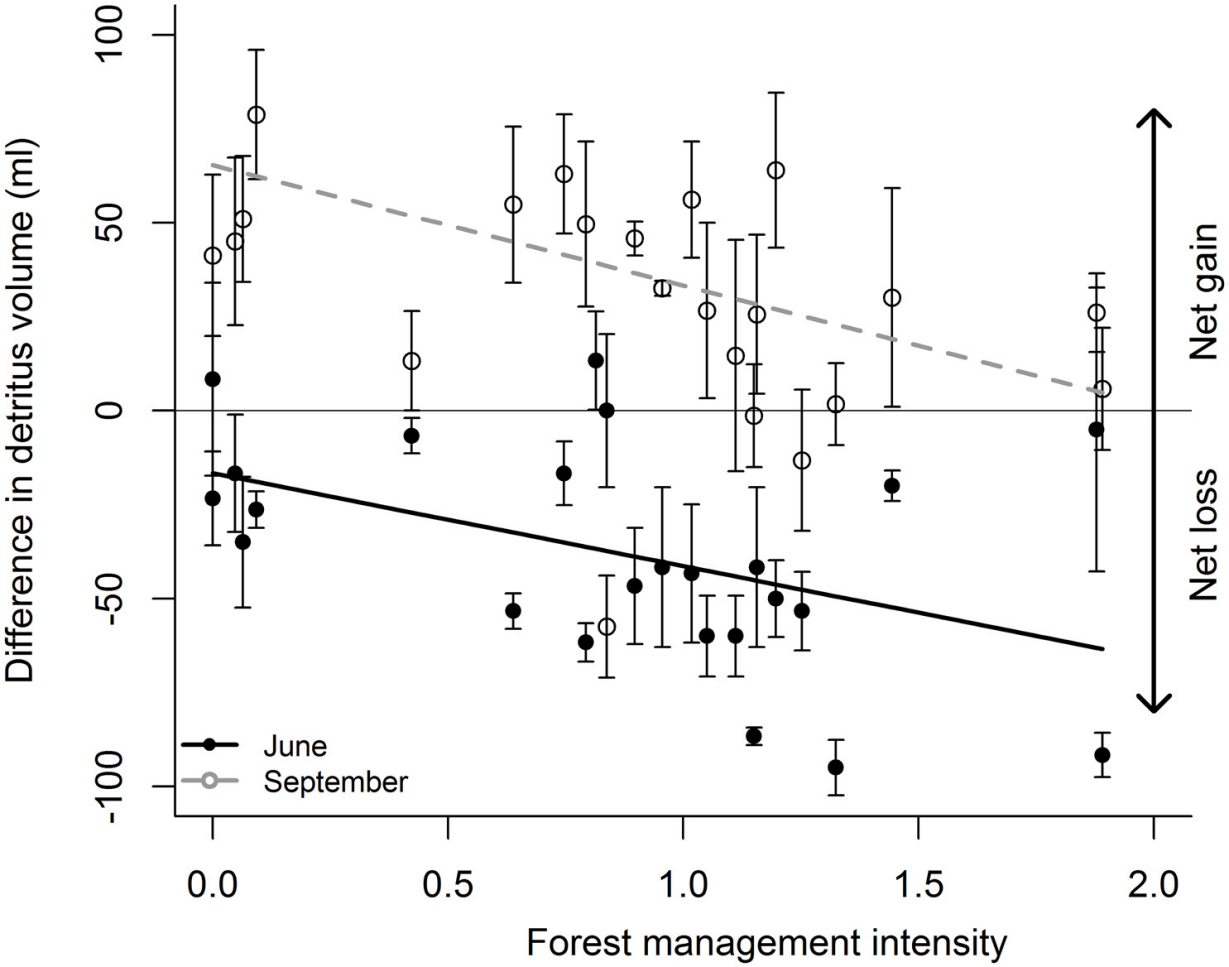
Effect of forest management intensity on difference in detritus volume. Difference in volume of detritus in the artificial tree holes was calculated between the start of the experiment and harvesting time (June or September). Values above zero represent a net gain, below zero a net loss of detritus from the tree hole. Data are from the Hainich region (S4 Table) from June (closed symbols and solid lines) or from September (open symbols and dashed line). Symbols represent means per plot, error bars show ±SE. Forest management intensity was calculated according to Kahl and Bauhus [30]. For results of statistical analyses see S4 Table.

Forest management affected detritus volume in the artificial tree holes at the end of the experiment (only measured in the Hainich): The higher the forest management intensity the lower the final volume of detritus in the artificial tree holes (Fig 4, S4 Table). Tree holes sampled in June contained less detritus than at the start of the experiment (net loss, Fig 4), a sign of decomposition occurring in the holes. By September, tree holes had—through natural leaf fall—acquired on average more detritus than they started off with (net gain, Fig 4). However, the negative effect of forest management on detritus volume was visible across the season. Organism abundance and detritus volume in the artificial tree holes at the time of sampling were positively related (S2 and S4 Tables).

The response of tree-hole nutrient and oxygen contents to forest management were tested, however, phosphate and nitrate content were only measured in the Hainich in June. The abundance of organisms in the holes had a positive influence on nutrient contents (except ammonium in the Alb; S2 and S3 Tables) whereas forest management decreased their content (except ammonium in the Alb) even after fitting species abundance and richness as covariates. Phosphate content was additionally negatively related to the density of natural tree holes in the plots. In the Hainich, ammonium content was negatively related to water temperature. Oxygen concentration was measured in the Hainich but was unrelated to any of the measured variables (S2 Table).

### Comparison between artificial and natural tree holes

The abiotic conditions differed between artificial and natural tree holes. Water temperature was only measured in the Hainich in June (at the same time as natural tree holes) where it was higher in artificial (mean±standard error = 17.6±0.5°) than in natural tree holes (15.7±0.5°C; F_1,45_ = 4.31, P = 0.0437 from a linear mixed model with plot as the random effect). Oxygen content was only measured in the Hainich and was higher in artificial (1.25±0.13mg/l) than in natural tree holes (1.00±0.09mg/l; F_1,36_ = 6.26, P = 0.0170 from a linear mixed model with plot as a random effect and square-root-transformed abundance of organism as a covariate).

**Fig 5:**
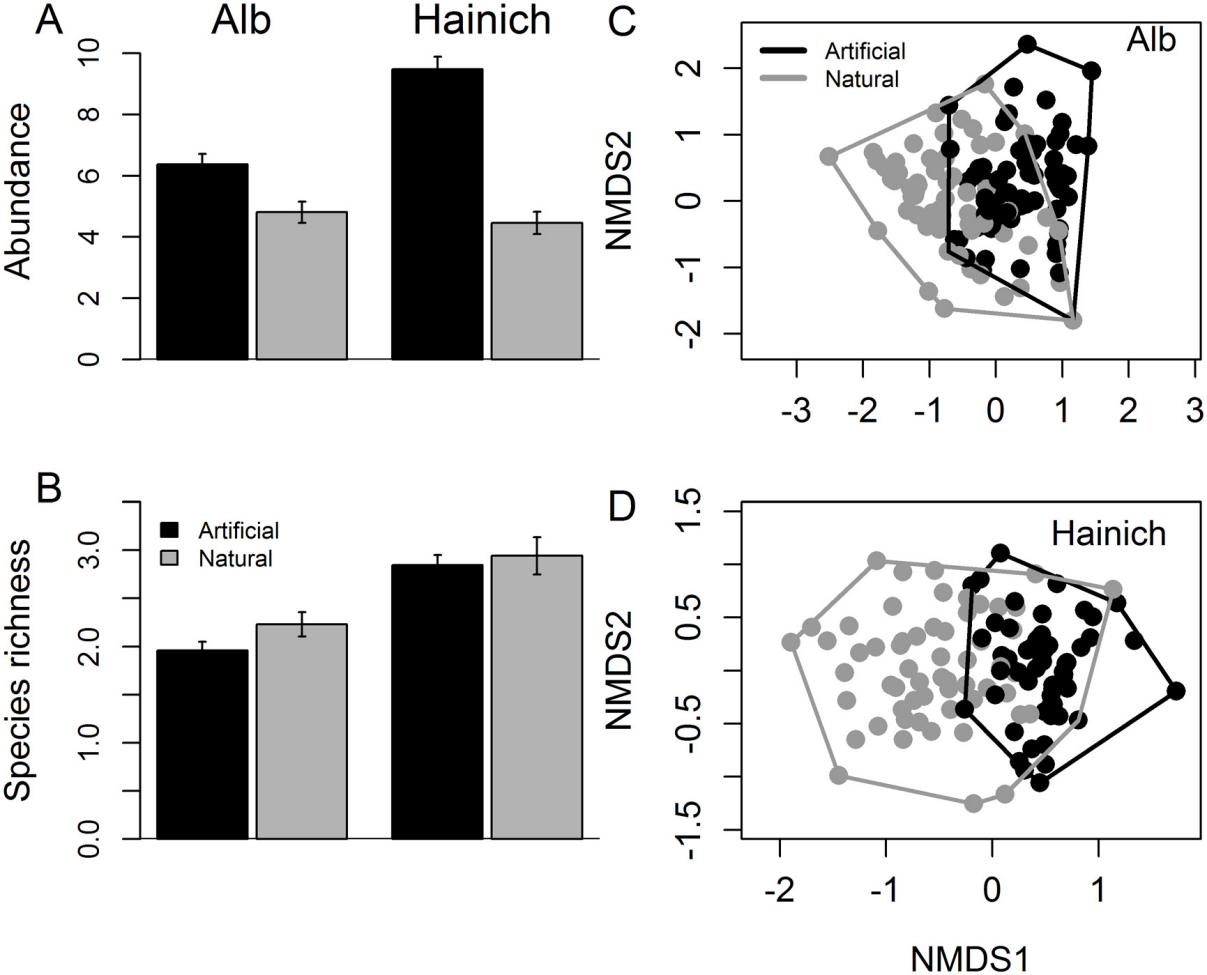
Comparison of macroinvertebrate communities of artificial and natural tree holes in the two regions in June. A) abundance (square-root transformed), B) species richness and C), D) composition. Artificial holes are depicted by black bars and symbols, natural holes by grey bars and symbols. Average water volume differed between artificial and natural tree holes (artificial tree holes had larger average volume). However, the differences in abundance and richness between artificial and natural tree holes were still significant after fitting water volume as a covariate (for results of statistical analyses see text). Raw abundance and species richness (i.e. not adjusted for differences in volume) are shown here. The low overlap between artificial and natural communities in the composition plots (C, D) shows that community composition differed between artificial and natural tree holes in both regions. Only taxa with more than five occurrences per region across both tree-hole types were used for the nonmetric multidimensional scaling (NMDS) plots in C and D. Stress = 0.17, n = 214 for Alb, stress = 0.21, n = 124, for Hainich. Please note that experimental set-up and the volume of artificial tree holes also varied between the two regions, so they cannot be compared directly.

The abundance of organisms (square-root transformed) was higher in artificial than natural tree holes (Fig 5A), even when the difference in water volume between the tree-hole types was taken into account (F_1,229_ = 19.34, P<0.001 for the Alb and F_1,154_ = 26.74, P<0.001 for the Hainich from two separate linear mixed models with plot as a random effect and water volume as a covariate). Species richness was lower in artificial than natural tree holes (Fig 5B), even when the difference in water volume and abundance between the tree-hole types was taken into account (F_1,228_ = 51.30, P<0.001 for the Alb and F_1,153_ = 13.44, P<0.001 for the Hainich from two separate linear mixed models with plot as a random effect and water volume and square-root transformed abundance as covariate).

Overall, community composition differed between artificial and natural tree holes in both regions (Fig 5C and 5D, F_1,213_ = 26.47, P<0.001 for the Alb, F_1,123_ = 21.98, P<0.001 for the Hainich). The four most abundant taxa in artificial tree holes (*Dasyhelea* sp., *Metriocnemus cavicola*, Cecidomyiidae sp. 1 and *Myathropa florea*) were also among the most frequent dwellers of natural tree holes [10]. However, natural tree holes harbored additional taxa at high frequencies that rarely occurred in artificial tree holes. The most striking differences was the almost complete absence of four species in our artificial tree holes: the beetle *Prionocyphon serricornis*, the Culicidae species *Aedes geniculatus*, an additional Culicidae species and a Chironomidae species, all of which frequently occurred in natural tree holes [10].

## Discussion

Overall, we found that forest management intensity indeed influenced communities in artificial tree holes. In particular, higher forest management intensity decreased abundance and richness, shifted community composition and decreased detritus content in these holes. The effect of forest management persisted across the season and was evident in both regions. Local environmental variables affected tree-hole communities in some cases, while the density of natural tree holes (as a proxy for dispersal opportunities) had surprisingly weak effects. Artificial tree holes differed from natural ones in environmental conditions, species abundance, richness and community composition. However, the negative management effects were detectable in both systems.

### Effects of forest management on communities, detritus content, oxygen and nutrient concentrations (Hypothesis 1)

Forest management intensity strongly affected the communities in artificial tree holes, supporting our first hypothesis. Specifically, abundance and richness were lower in forests with higher intensity of management across both regions and according to both management intensity indices (ForMI and SMI). While the communities showed a strong dominance of four taxa (*Dasyhelea* sp., *Metriocnemus cavicola*, Cecidomyiidae sp. 1 and *Myathropa florea*), it was not those most abundant species that were driving the effect. We found those abundant species to be affected by few of the variables measured in our study (see below), potentially because their habitat requirements were generally met. However, not much is known on habitat preferences of these species [9]. Instead of specific responses of certain species, the community as a whole responded to management with shifts in total abundance, species richness and composition. This change in community structure is in line with the few studies on the effects of land-use intensity on invertebrate communities in tree holes or artificial analogues [10, 26, 27, 48]. The strength of the effect of management intensity on tree-hole communities in our study suggests that they are one of the more strongly affected communities in temperate forests and may indeed be used as indicator systems to detect changes in natural communities as a response to management decisions.

We furthermore found that the negative management effects on species richness persisted across the season. However, we did not detect a management effect on abundance in September. Abundance was on average higher in September and species composition in terms of the most dominant species shifted across the season. These changes across the season are in line with partly asynchronous seasonal fluctuations of individual species found by Ptatscheck and Traunspurger [22], although in their study overall fluctuations of insect larvae appeared to be synchronous. The species-specific fluctuations may reflect different colonization patterns or life cycles of the species. However, there is little information available on the life-history characteristics of the species in our study.

Decomposition of dead plant material (detritus) is one of the major ecosystem functions in tree-hole systems. Tree holes sampled in June showed a net loss of detritus while by September, tree holes had gained more detritus than was experimentally added at the start. Nevertheless, the negative effect of forest management on detritus volume was evident across the season. With our experimental design we are unable to separate the contrasting effects of decomposition and litter input. However, the maximum net loss of detritus is quite substantial, which suggests that the reason for the different detritus amounts at the end of the experiment in different management intensities is not only due to differential litter input. Decomposition could be higher in high management intensities due to differences in litter quality [49]. In forests under intensive management, lower tree species richness (i.e. here mostly beech) and a more even age structure might result in more uniform litter that may be easier to decompose [50]. Phosphate and nitrate contents were lower at higher management intensity. With higher decomposition we would have expected the opposite due to increased release of nutrients from the decomposed leaf litter. Here, shifts in species composition with higher forest management intensity or different densities of tree holes might potentially explain the different decomposition processes. Again, differences in litter quality could also have played a role. Additionally, nutrients could originate from stemflow instead of decomposing plant material or from other inputs such as droppings of birds [51] which were regularly observed in tree holes (pers. observation). However, data on nutrient contents of stemflow available for certain plots in our study (Martin T. Schwarz, pers. comm.) did not show any relationship with phosphate, nitrate or ammonium contents in the tree holes (data not shown).

### Effects of the local environment and dispersal limitation (Hypothesis 2)

We found effects of certain local environmental conditions on tree-hole communities, partly confirming our second hypothesis. For example, the type of opening of the tree holes (top vs. side) which we experimentally manipulated in our study had an effect on invertebrate abundance and richness. Holes which opened at the top were probably more easily found by colonizing organisms and received more rain water and detritus input (indicated by data from this study and Gossner et al. [10]). Therefore, they might have reached a higher abundance of inhabiting organisms and, irrespective of abundance, also greater species richness. However, management effects were detectable in artificial holes with both opening types. Only one of the four most abundant species showed a clear preference: *Myathropa florea* was more abundant in artificial tree holes with a top opening than with a side opening. Schmidl et al. [9] also report that *M*. *florea* prefers shallow, open holes. On the other hand, vertically opening holes are typically preferred by drought-sensitive species [52], likely some of our mosquito species. However, mosquito numbers were low in our study and a preference for a hole opening type did not emerge.

The diameter of the tree that the artificial holes were attached to was found to have a non-consistent effect on the abundance of organisms and a positive effect on richness (when forest management intensity was fitted last in the model) in the Hainich (not measured in the Alb). A larger tree might affect stem flow and thus water volume and nutrients. It might also reflect the higher quality of the habitat directly surrounding the tree hole (e.g. shelter for adults) which might be important for some species but not for all. In age class forests this variable likely describes a larger area (since all trees are of the same age and thus, have a similar diameter). However, in selection cutting or unmanaged forests, variability in tree ages and sizes is much higher and this variable indeed only describes the direct surrounding of the tree hole. The relevant scale for each species will depend on their dispersal abilities and behavior.

As an indicator of potential dispersal limitation the density of natural tree holes in our plots had a rather weak influence on the communities in artificial tree holes. If species had been dispersal limited, then a higher density of natural tree holes (i.e. a higher density of breeding sites or sources of colonizers) would have resulted in higher abundance or species richness in our tree holes. However, only when forest management intensity was fitted last in the model, a positive effect of the density of natural holes on the abundance of tree-hole organisms appeared and partially explained the negative management effect, indicating a certain amount of dispersal limitation due to a lower density of tree holes in forests with higher management intensity, an effect which we showed previously [10]. Average densities of natural tree holes did not differ significantly between the regions. There were 15±3 natural water-filled tree holes per ha in the Alb and 19±3 per ha in the Hainich (range 0–50 per ha in the Alb and 0–56 per ha in the Hainich [10]).

A potential alternative reason for the weak effects of tree-hole density on our communities could be that other aquatic habitats were available in the plots or in the surrounding area so that species did not depend on tree holes as exclusive breeding grounds. Since most of the species found in our study are indeed habitat generalists and not restricted to breeding in tree holes, this is generally possible. We did not quantify the presence or density of other aquatic habitats in the plots. However, they were relatively scarce and limited to small puddles or tracks by forestry machinery (pers. obs.). Therefore, tree holes appear to be an important source of water in these sites for organisms with aquatic breeding habitats and the effect of natural tree-hole density on our communities is likely a valid indicator of dispersal limitation. Community composition in artificial holes was indeed affected by the density of natural tree holes in the Alb, suggesting dispersal limitations in at least some species. Generally, it seems, however, that dispersal is not limiting for the majority of the species in tree holes in our study, though. In a study on natural tree holes in the same area we similarly found little evidence of dispersal limitation in these species [10]. Only for one of the four most abundant species in our study, Cecidomyiidae sp. 1, we did find a strong positive effect of tree-hole density on its average abundance in artificial tree holes in the Alb. Members of this family can be predacious and it has been shown previously that predators are more strongly affected by small habitat size and low dispersal possibilities [53–55] likely because they have small population sizes and need their prey to arrive first. However, the generally high abundance of this species does not support their role as an obligate predator in the tree-hole food web. Overall, we did not find many species that we can unequivocally classify as predators. This low number of predatory species has been described previously from natural temperate tree holes [10, 56]. We hypothesize that external terrestrial predators may play a role in controlling the abundances and species composition in artificial and natural tree holes and that some differences between management intensities might stem from this (as yet unmeasured) cross-system top-down control.

In addition to effects of measured environmental conditions, differences in climate and in maximum management intensity between the study regions could have affected the response of certain species. Furthermore, differences in experimental set-up between the study sites could have had an influence. For example, the differences in the sizes of the artificial tree holes between the two regions could have resulted in a different ability to detect environmental effects on certain species. Unfortunately, information on larval habitat preferences of the species in our study is extremely scarce.

### Environmental conditions and communities in artificial vs. natural tree holes (Hypothesis 3)

We found that artificial and natural tree holes in the same area differed in abiotic conditions as well as in inhabiting communities. Specifically, we found that artificial tree holes had higher temperature and oxygen concentrations. Of course, part of these differences in abiotic and biotic conditions may simply result from our choice of containers and container sizes to serves as artificial tree holes. In terms of their inhabiting organisms, artificial tree holes had higher overall species abundance (irrespective of differences in water volume), lower species richness and different community compositions. Certain species exclusively occurred in natural tree holes in our study area. Two of these species–*Prionocyphon serricornis* (Coleoptera) and *Aedes geniculatus* (Culicidae)–were already reported by Rohnert [7] to be associated with natural tree holes and to rarely occur in other habitats. In the case of the beetle, exposure time of the artificial tree holes may have been too short since *P*. *serricornis* have long larval periods of up to two years [57]. *A*. *geniculatus* has been reported to prefer a certain combination of environmental conditions [9], which was likely not met in our artificial tree holes.

### Artificial vs. natural tree holes as detectors of change (Hypothesis 4)

Despite the abiotic and biotic differences between artificial and natural tree holes, negative management effects on abundance and species richness were detected in communities in both hole types. These results suggest that artificial tree holes may be a useful experimental model system to identify and measure change in forest ecosystems under changing management. Previously, Lounibos [58] found that artificial tree-hole analogues may show different relative abundances of species than natural ones but still represent natural communities to a large degree. Pimm and Kitching [59] propose that experimental conditions in artificial tree-hole analogues do imply a disturbance to communities but one that is comparable to natural drought or winter freezing conditions. Yanoviak [49, 60] and Yanoviak and Fincke [28] suggest that despite differences between artificial and natural tree holes in certain abiotic parameters and species richness, tree-hole analogues are suitable to test ecological effects on aquatic communities. Our results support this notion. In addition, we would recommend the use of artificial tree holes in conservation assessments, for example for before-after comparisons of forest habitats to quantify effects of management measures or to evaluate differences between differently managed forests. Here, both natural and artificial tree holes could serve as a simple indicator systems allowing a potentially rapid detection of changes in response to management. However, artificial tree-hole analogues offer cheap and simple solutions that can be easily replicated. The containers can be fixed to trees at manageable heights and do not require any additional maintenance (except during drought conditions). Assembled communities can be collected after a few months of exposure. After this relatively short period of time, effects of forest management on species richness and abundance should already be detectable.

## Conclusions

Our results indicate that forest management has substantial and pervasive effects on communities which may alter their structure and functioning through a number of different mechanisms. Whereas more direct impacts of management on other types of communities in forests had been reported before [1, 3–5], the effect on aquatic communities constitutes novel information (but see Gossner et al. [10] for natural tree holes). We did not find strong individual direct pathways that may explain the effect of forest management intensity on communities via the measured environmental variables. Instead, multiple indirect effects seem to be central here. These could for example operate via effects of management on the species richness of trees and other plants, changing light and temperature conditions, resource input into tree holes and the structure of the matrix between communities that may influence dispersal processes and conditions for adult insects (e.g. nutrition and shelter). By investigating community change in tree holes we may be integrating over a number of direct and indirect effects to derive a more comprehensive picture of the consequences of change in forest management intensity on communities. We furthermore conclude that artificial tree-hole analogues constitute a useful experimental alternative to test effects of land-use change on natural communities and may indeed be used as an early-warning or indicator system of ecosystem modifications in conservation assessments.

## Supporting Information

S1 Fig Pictures of natural tree holes in the study plots. The two pictures on the left show pans, the two on the right show rot holes in branch breaks. Photo credits: M. M. Gossner.
(TIF)

S2 Fig Pictures of the artificial tree holes used in this study. The two pictures on the left show the artificial tree holes in the Alb, which had a maximum volume of 10l and either a side opening (left) or a top opening (second from left). The two pictures on the right show the artificial tree holes used in the Hainich with a maximum volume of 600ml. For further information on tree-hole characteristics and measurements in the two regions, see Fig 1 and S1 Table. Photo credits: left: A. Rohland, others: P. Lade.
(TIF)

S3 Fig Pictures of plots in the Alb along increasing forest management intensity. From top left to bottom right, the Forest Management Index (ForMI) increases from 0.00, 0.90, 1.19, 1.57 and 2.35 to 2.82. Forest management intensity was calculated according to Kahl and Bauhus [30]. Photo credits: M. Fellendorf.
(TIF)

S4 Fig Pictures of plots in the Hainich along increasing forest management intensity. From top left to bottom right, the Forest Management Index (ForMI) increases from 0.00, 0.64, 0.83, 0.96 and 1.11 to 1.88. Forest management intensity was calculated according to Kahl and Bauhus [30]. Photo credits: top row and bottom left: M. M. Gossner, bottom middle and left: Institute of Ecology, University of Jena.
(TIF)

S5 Fig Average abundance of the different taxa per tree hole. Abundance was square-root transformed and is shown for the Alb region in June and in the Hainich region in June and September. Many of the taxa were not identified to species level but classified as morphospecies. Family names given on the left (except for “Brachycera” species which were not determined to family level) Error bars are omitted for better readability.
(TIFF)

S1 Methods Measurement of nutrient contents. (DOCX)

S1 Table Artificial tree-hole characteristics and measurements. (DOCX)

S2 Table Mixed model results for Hainich (June). (DOCX)

S3 Table Mixed model results for ammonium in the Alb (June). (DOCX)

S4 Table Mixed model results for detritus in the Hainich (June and September). (DOCX)

S5 Table Mixed model results for individual species in the Alb (June) and in the Hainich (June and September). (DOCX)
