# Supplementary figures and images for: Forest Management Intensity Affects Aquatic Communities in Artificial Tree Holes
Source: PLoS One. 2016 May 17;11(5):e0155549. doi: 10.1371/journal.pone.0155549 (PMC4871352; doi:10.1371/journal.pone.0155549)

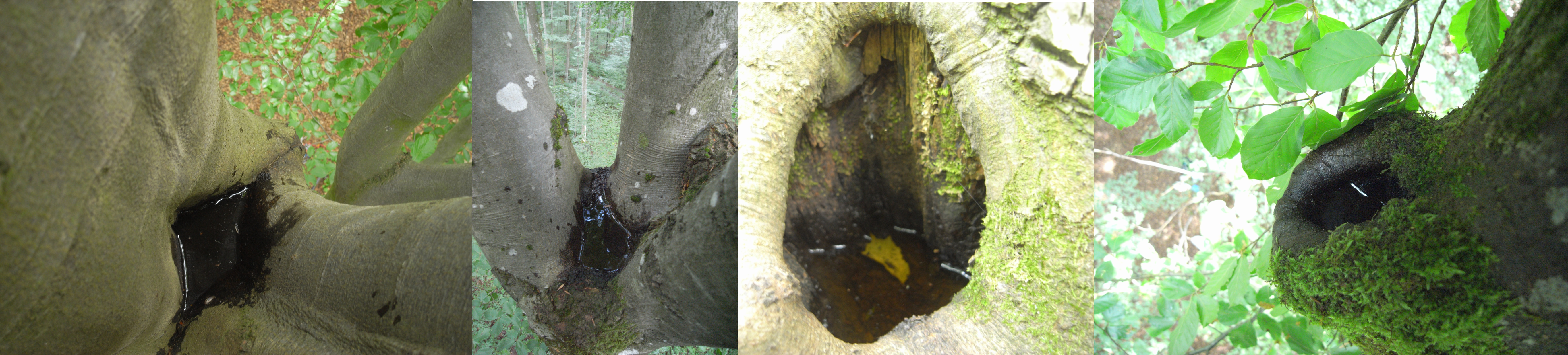

Supplement: S1 Fig — The two pictures on the left show pans, the two on the right show rot holes in branch breaks. Photo credits: M. M. Gossner. (TIF) [file pone.0155549.s001.tif]

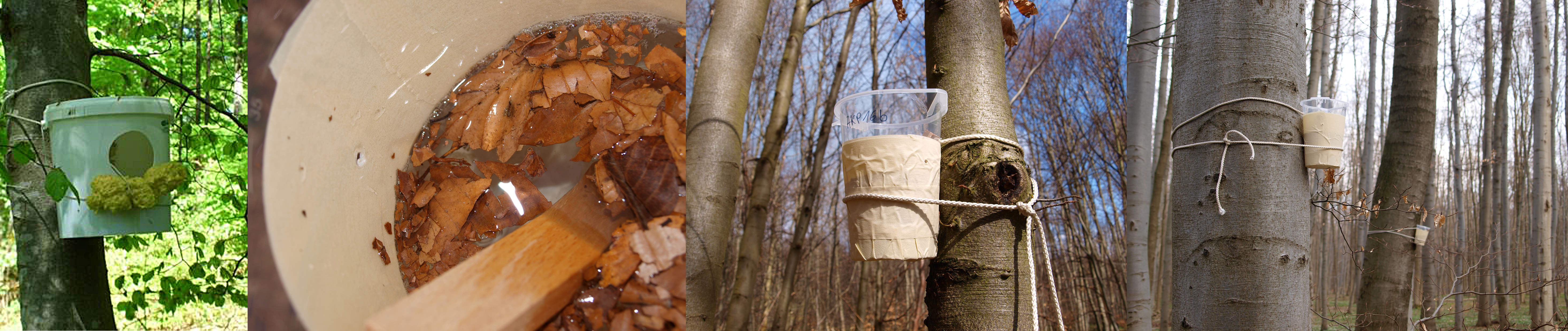

Supplement: S2 Fig — The two pictures on the left show the artificial tree holes in the Alb, which had a maximum volume of 10l and either a side opening (left) or a top opening (second from left). The two pictures on the right show the artificial tree holes used in the Hainich with a maximum volume of 600ml. For further information on tree-hole characteristics and measurements in the two regions, see Fig 1 and S1 Table. Photo credits: left: A. Rohland, others: P. Lade. (TIF) [file pone.0155549.s002.tif]

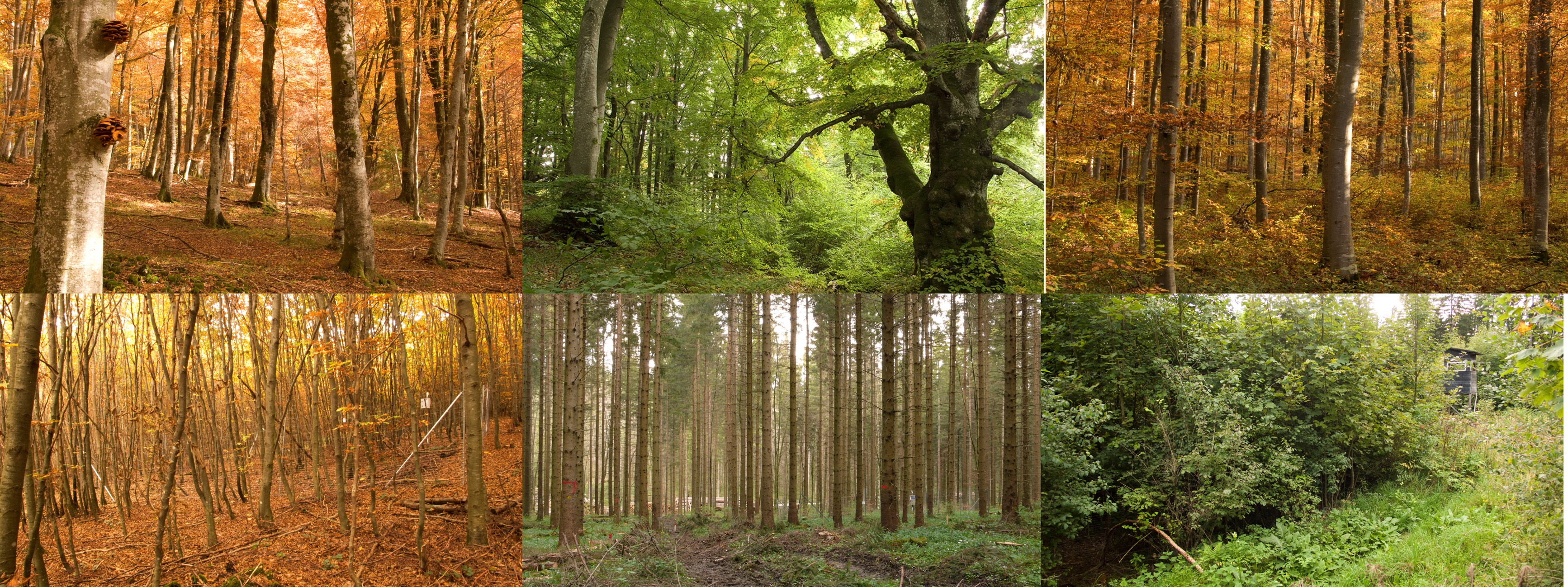

Supplement: S3 Fig — From top left to bottom right, the Forest Management Index (ForMI) increases from 0.00, 0.90, 1.19, 1.57 and 2.35 to 2.82. Forest management intensity was calculated according to Kahl and Bauhus [30]. Photo credits: M. Fellendorf. (TIF) [file pone.0155549.s003.tif]

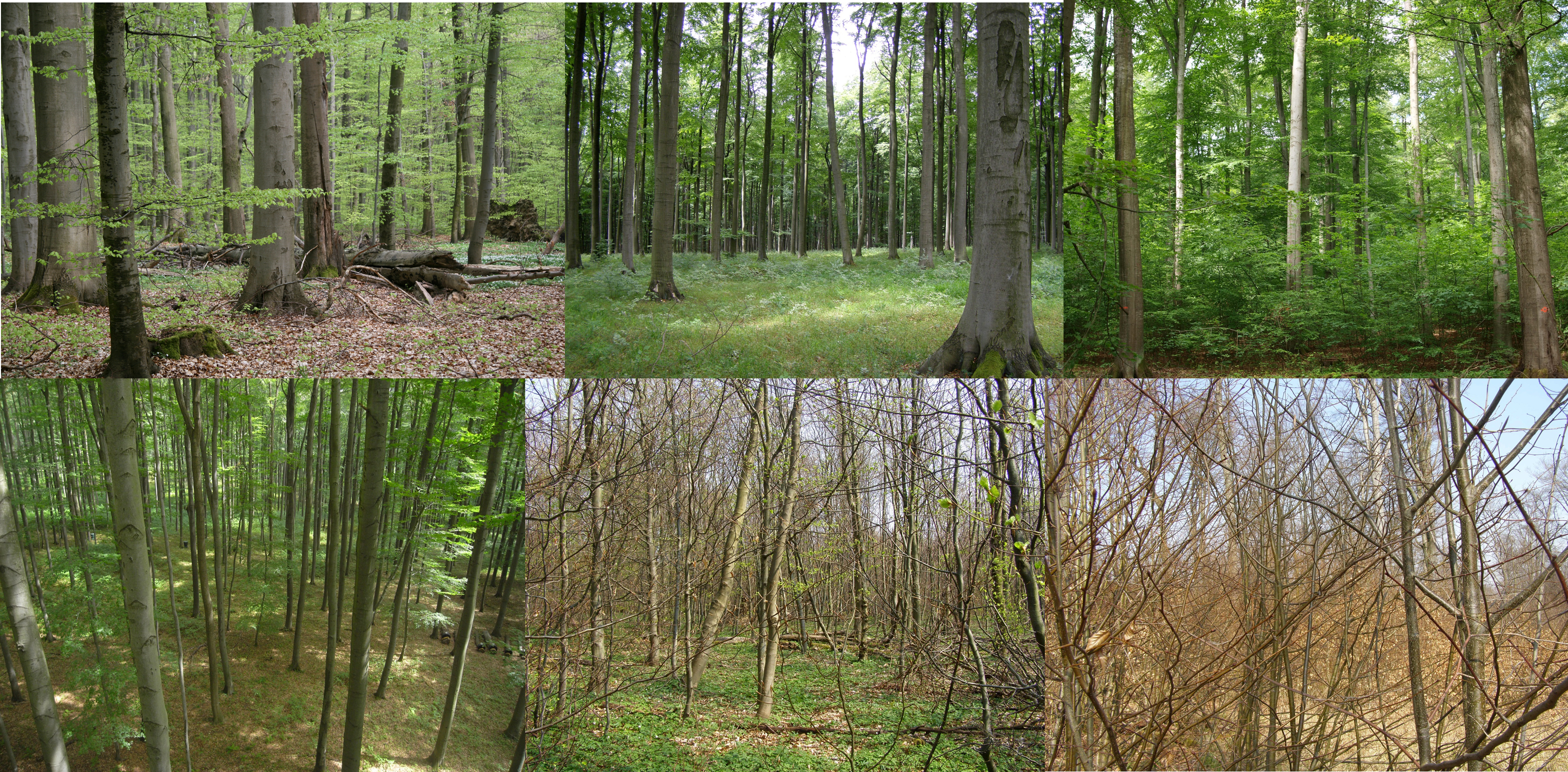

Supplement: S4 Fig — From top left to bottom right, the Forest Management Index (ForMI) increases from 0.00, 0.64, 0.83, 0.96 and 1.11 to 1.88. Forest management intensity was calculated according to Kahl and Bauhus [30]. Photo credits: top row and bottom left: M. M. Gossner, bottom middle and left: Institute of Ecology, University of Jena. (TIF) [file pone.0155549.s004.tif]

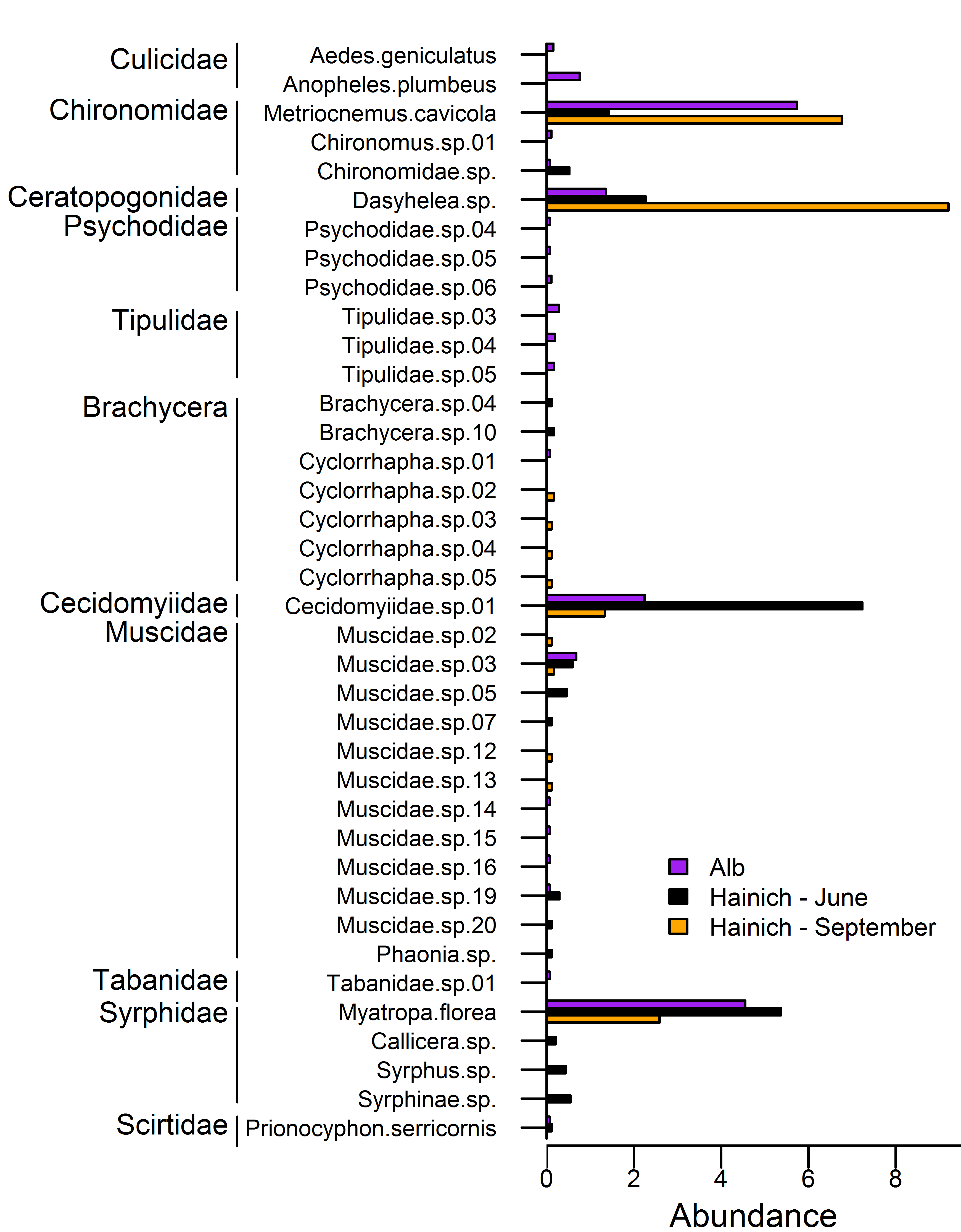

Supplement: S5 Fig — Abundance was square-root transformed and is shown for the Alb region in June and in the Hainich region in June and September. Many of the taxa were not identified to species level but classified as morphospecies. Family names given on the left (except for “Brachycera” species which were not determined to family level) Error bars are omitted for better readability. (TIFF) [file pone.0155549.s005.tiff]
