# Supplementary material for: Forest Management Intensity Affects Aquatic Communities in Artificial Tree Holes
Source: PLoS One. 2016 May 17;11(5):e0155549. doi: 10.1371/journal.pone.0155549 (PMC4871352; doi:10.1371/journal.pone.0155549)
Supplement: S1 Methods — (DOCX) [file pone.0155549.s006.docx]

**S1 Methods: Measurement of nutrient contents**

Nitrate-, ammonium- and phosphate contents were measured photometrically. For the measurement of nitrate content 1-ml subsamples were diluted if necessary and mixed with 50μl of 5-% resorcin solution and 1.3ml of concentrated acid sulfur. After mixing and heat dissipation an absorbance measurement was taken in a 1.5-ml cuvette at 360nm. For the colorimetrical measurement of ammonium, 1-ml subsamples of each sample were charged with 0.5ml of a natrium-phenolate solution, 0.25ml natrium-nitroprussid and natrium-hypochlorite-NaOH solution. The test tubes were subsequently stored in a water quench in the dark at 30°C for 30 minutes. Then, the absorbance measurements were taken in a 1.5-ml cuvette at 630nm. For the measurement of the phosphate concentration 2.5ml of each water sample were charged with 100 μl of molybdat-reagent solution and 25μl of ascorbic-acid solution and mixed. After a settlement period of 20 minutes an absorbance measurement was taken in a 1.5-ml cuvette at 880nm.
