# Supplementary material for: Forest Management Intensity Affects Aquatic Communities in Artificial Tree Holes
Source: PLoS One. 2016 May 17;11(5):e0155549. doi: 10.1371/journal.pone.0155549 (PMC4871352; doi:10.1371/journal.pone.0155549)
Supplement: S1 Table — (DOCX) [file pone.0155549.s007.docx]

**S1 Table. Artificial tree-hole characteristics and measurements in the two regions, Alb and Hainich.** Total number signifies the number of tree holes set up in each region at six tree holes per plot. Upper and lower diameter (mm), height (mm) and max. volume (l) refer to the container size. Water and detritus were added at the start of the experiments. Opening type was manipulated in the Alb (see Fig. 1). Forest management intensity was calculated according to Kahl and Bauhus [1]. Tree-hole density describes the number of natural tree holes per plot. Volume is the final water volume in each tree hole measured in ml. Tree diameter was measured at breast height in cm. Temperature is water temperature measured in °C. Abundance and richness was recorded by counting the individuals per tree hole and identifying them to morphospecies. Detritus was assessed as final detritus volume (in ml). Phosphate, nitrate and ammonium content were measured in in mg/l and oxygen concentration in %.

1. Kahl T, Bauhus J. An index of forest management intensity based on assessment of harvested tree volume, tree species composition and dead wood origin. Nat Conserv. 2014;7:15-27. doi: 10.3897/natureconservation.7.7281.
