# Supplementary material for: Forest Management Intensity Affects Aquatic Communities in Artificial Tree Holes
Source: PLoS One. 2016 May 17;11(5):e0155549. doi: 10.1371/journal.pone.0155549 (PMC4871352; doi:10.1371/journal.pone.0155549)
Supplement: S2 Table — (DOCX) [file pone.0155549.s008.docx]

**S2 Table. Mixed model results for Hainich (June).** Results from linear mixed models testing the effect of forest management intensity and a number of environmental variables on the abundance (square-root transformed) and species richness of communities, as well as on detritus volume (ml) and phosphate, nitrate and ammonium content (in mg/l) and oxygen concentration (in %) in artificial tree holes in the Hainich region in June 2011. Abundance (square-root transformed) is used as a covariate in the analysis of richness, and abundance (square-root transformed) and richness are used as covariates in all other analyses. Forest management intensity was calculated according to Kahl and Bauhus [1].Tree-hole density describes the number of natural tree holes per plot. Tree diameter was measured at breast height in cm. P-values<0.05 are printed in bold. For significant continuous main effects the direction of the effect is given: ↑ positive, ↓ negative. ndf: numerator degrees of freedom, ddf: denominator degrees of freedom.

1. Kahl T, Bauhus J. An index of forest management intensity based on assessment of harvested tree volume, tree species composition and dead wood origin. Nat Conserv. 2014;7:15-27. doi: 10.3897/natureconservation.7.7281.
