# Supplementary material for: Forest Management Intensity Affects Aquatic Communities in Artificial Tree Holes
Source: PLoS One. 2016 May 17;11(5):e0155549. doi: 10.1371/journal.pone.0155549 (PMC4871352; doi:10.1371/journal.pone.0155549)
Supplement: S3 Table — (DOCX) [file pone.0155549.s009.docx]

**S3 Table. Mixed model results for ammonium in the Alb (June).** Results from a linear mixed models testing the effect of forest management intensity and a number of environmental variables on ammonium content (mg/l) in the Alb in June. Abundance (square-root transformed) and richness of tree-hole communities are used as covariates in the analysis. Forest management intensity was calculated according to Kahl and Bauhus [1]. Tree-hole density describes the number of natural tree holes per plot. Artificial tree holes with two different opening types (top vs. side) were used. Volume is the final water volume of artificial tree holes in ml. ndf: numerator degrees of freedom, ddf: denominator degrees of freedom.

1. Kahl T, Bauhus J. An index of forest management intensity based on assessment of harvested tree volume, tree species composition and dead wood origin. Nat Conserv. 2014;7:15-27. doi: 10.3897/natureconservation.7.7281.
