# Supplementary material for: Forest Management Intensity Affects Aquatic Communities in Artificial Tree Holes
Source: PLoS One. 2016 May 17;11(5):e0155549. doi: 10.1371/journal.pone.0155549 (PMC4871352; doi:10.1371/journal.pone.0155549)
Supplement: S4 Table — (DOCX) [file pone.0155549.s010.docx]

**S4 Table. Mixed model results for detritus in the Hainich (June and September).** Results from a linear mixed models testing the effect of forest management intensity and a number of environmental variables on detritus volume (ml) in the Hainich in June and September. Abundance (square-root transformed) and richness of tree-hole communities are used as covariates in the analysis. Forest management intensity was calculated according to Kahl and Bauhus [1].Tree-hole density describes the number of natural tree holes per plot. Time refers to data collection in June vs. September. Tree diameter was measured at breast height in cm. P-values<0.05 are printed in bold. For significant continuous main effects the direction of the effect is given: ↑ positive, ↓ negative. ndf: numerator degrees of freedom, ddf: denominator degrees of freedom.

1. Kahl T, Bauhus J. An index of forest management intensity based on assessment of harvested tree volume, tree species composition and dead wood origin. Nat Conserv. 2014;7:15-27. doi: 10.3897/natureconservation.7.7281.
