# Supplementary material for: Forest Management Intensity Affects Aquatic Communities in Artificial Tree Holes
Source: PLoS One. 2016 May 17;11(5):e0155549. doi: 10.1371/journal.pone.0155549 (PMC4871352; doi:10.1371/journal.pone.0155549)
Supplement: S5 Table — (DOCX) [file pone.0155549.s011.docx]

**S5 Table. Mixed model results for individual species in the Alb (June) and in the Hainich (June and September).** Results from linear mixed models testing the effect of forest management intensity and a number of environmental variables on the abundance (square-root transformed) of the four most abundant species in artificial tree holes in the two regions Alb and Hainich. Forest management intensity (ForMI) was calculated according to Kahl and Bauhus [1].Tree-hole density describes the number of natural tree holes per plot. In the Alb, artificial tree holes with two different opening types (top vs. side) were used. Volume is the final water volume of artificial tree holes in ml. Time refers to data collection in June vs. September (September data is only available for the Hainich). Tree diameter was measured at breast height in cm. P-values<0.05 are printed in bold. For significant continuous main effects the direction of the effect is given: ↑ positive, ↓ negative. ndf: numerator degrees of freedom, ddf: denominator degrees of freedom.

1. Kahl T, Bauhus J. An index of forest management intensity based on assessment of harvested tree volume, tree species composition and dead wood origin. Nat Conserv. 2014;7:15-27. doi: 10.3897/natureconservation.7.7281.
